# Euglycemic Diabetic Ketoacidosis Mimicking an Acute Asthma Exacerbation in a Young Adult With New‐Onset Diabetes: A Case Report

**DOI:** 10.1002/ccr3.73613

**Published:** 2026-09-28

**Authors:** Faizan E Mustafa, Sarim Hassan Shahab, Basit Ali, Aleena Majeed, Fazal Habib, Muhammad Sajjad

**Affiliations:** ^1^ Nishtar Medical University Multan Pakistan; ^2^ Advent Health Orlando Florida USA; ^3^ Sher‐e‐Bangla Medical College Barisal Bangladesh; ^4^ Nowshera Medical College Nowshera Pakistan

**Keywords:** diabetes mellitus, euDKA, euglycemic diabetic ketoacidosis, high‐anion‐gap metabolic acidosis, ketogenic diet, low‐carbohydrate diet, Type 1 diabetes mellitus

## Abstract

Euglycemic diabetic ketoacidosis can mimic acute asthma, including in previously undiagnosed diabetes and without SGLT‐2 inhibitor use. Carbohydrate restriction may precipitate life‐threatening ketoacidosis, particularly with insulin deficiency. Early arterial blood gas analysis and urine ketone testing facilitate prompt diagnosis, enabling timely treatment and preventing complications.

## 1 Introduction

Diabetic ketoacidosis (DKA) is traditionally described as a triad of severe hyperglycemia (blood glucose level > 250 mg/dL), metabolic acidosis, and ketonemia [1]. A well‐recognized but underdiagnosed variant, known as euglycemic DKA (euDKA), is characterized by a blood glucose level below 200 mg/dL in the presence of significant ketonemia or ketonuria and metabolic acidosis, as outlined in recent international consensus recommendations [2, 3]. The diagnosis of euDKA is difficult because the only characteristic feature of severe hyperglycemia is often subtle or absent, and the condition can be easily overlooked during the initial evaluation. It was initially described by Munro et al. in 1973 [4]. euDKA is known to be triggered by a low‐carbohydrate diet, prolonged fasting, pregnancy, alcohol consumption, insulin use before presentation, chronic liver disease, glycogen storage diseases, and sodium‐glucose cotransporter‐2 (SGLT‐2) inhibitors [5, 6]. Most significantly, euDKA may also be the initial presentation of DM, particularly in young patients, who may have no classic hyperglycemic symptoms like polyuria, polydipsia, and polyphagia, further delaying diagnosis.

We report a case of a 19‐year‐old male with no prior known diabetes presenting to the ED with respiratory complaints that were initially thought to be due to asthma but subsequently diagnosed as euDKA. The episode was precipitated by a self‐imposed low‐carbohydrate diet that was undertaken to prepare for a competitive football tournament, in the absence of SGLT‐2 inhibitor use. The novelty of this case lies in the presentation of euDKA in a young adult with previously undiagnosed diabetes, without SGLT‐2 inhibitor exposure, following a very low‐carbohydrate diet. Similar cases have been reported, including a recent case by Helvaci et al. [7], but euDKA precipitated by carbohydrate restriction alone remains infrequently described. This case highlights the need to maintain a broad differential diagnosis when the patient is not responding to the initial treatment as expected, and to obtain arterial blood gas and urine ketone levels early in this type of clinical scenario.

## 2 Case Presentation

### 2.1 Case History and Initial Presentation

A 19‐year‐old male presented to the ED with nausea, mild difficulty breathing, restlessness, and drowsiness, with a previous clinical history of mild intermittent asthma, diagnosed in adolescence (confirmed by spirometry), with infrequent exacerbations managed on an as‐needed basis with an albuterol inhaler, and no prior hospitalizations or ED visits for asthma. His mother had a personal history of Type 1 diabetes mellitus; there was no history of diabetes in other family members. He was not on any medications besides his inhaler. His mother reported no history of polyuria, polydipsia, or polyphagia (excessive hunger) or any recent illness. His vitals included a pulse rate of 112 beats per minute, a respiratory rate of 25 breaths per minute, a blood pressure of 108/70 mmHg, an oxygen saturation (SpO_2_) of 93% on room air, and a blood sugar reading (BSR) of 164 mg/dL. His BMI (body mass index) was 18.7 kg/m^2^, and his Glasgow Coma Scale (GCS) score was 14/15. There was no abnormality on physical examination other than wheezing on auscultation of the chest. With the clinical picture of mild hypoxia and wheezing, and no significantly raised blood sugar, oxygen was commenced on a working diagnosis of acute exacerbation of asthma. His SpO_2_ improved to 98%, and he was kept under observation.

### 2.2 Clinical Deterioration and Investigations

The patient's condition started to deteriorate about 2 h later. He complained of abdominal pain and vomited twice. He became drowsy, his breathing was shallow, and his SpO_2_ dropped to 95%. The blood glucose was rechecked and was 212 mg/dL during deterioration and subsequently measured 198 mg/dL when euDKA was formally diagnosed. Arterial blood gas (ABG) analysis, urine ketones, and glycated hemoglobin (HbA1c) were obtained urgently in view of clinical deterioration. Full laboratory findings, with corresponding reference ranges, are presented in Table 1.

**TABLE 1:** Laboratory findings on presentation with reference ranges.

Parameter	Result	Reference range
Blood glucose	198 mg/dL	70–140 mg/dL (random)
pH	7.01	7.35–7.45
Bicarbonate	14 mEq/L	22–28 mEq/L
Anion gap	19 mEq/L	Usually 8–12 mEq/L without K^+^; some laboratories use ~7–16
Lactate	1.34 mmol/L	0.5–2.2 mmol/L
HbA1c	8.9%	< 5.7% is normal; 5.7%–6.4% prediabetes; ≥ 6.5% diabetes
Sodium	133 mEq/L	135–145 mEq/L
Chloride	100 mEq/L	98–107 mEq/L
Potassium	3.9 mEq/L	3.5–5.1 mEq/L
BUN	12 mg/dL	7–20 mg/dL
Creatinine	1.1 mg/dL	0.7–1.3 mg/dL
Hemoglobin	13 g/dL	Adult male commonly ~13.5–17.5 g/dL; adult female ~12–15.5 g/dL
Serum insulin	2.6 mU/L	2.6–24.9 mU/L (fasting insulin reference ranges are highly assay/lab dependent)
Fasting C‐peptide	0.39 ng/mL	0.81–3.85 ng/mL

The ABGs showed marked acidemia with high‐anion‐gap metabolic acidosis, and the urine dipstick was positive for ketones. Serum β‐hydroxybutyrate, the preferred quantitative marker of ketoacidosis, was not measured due to unavailability at our facility; ketosis was therefore assessed using urine ketone testing alone. HbA1c was consistent with chronic hyperglycemia over the preceding approximately 2–3 months. Serum lactate was normal, suggesting that the raised anion gap was not due to significant lactic acidosis. Renal function was preserved, and potassium and chloride levels were within normal limits. There was no clinical or biochemical evidence to suggest a renal or infectious cause of the acidosis. Both serum insulin and fasting C‐peptide were low, suggesting previously undiagnosed diabetes mellitus with reduced insulin secretion. Toxic ingestion, including alcohol and salicylates, was considered clinically unlikely based on the patient's history, although confirmatory serum levels were not obtained. Hypothyroidism was excluded due to normal thyroid function tests. No infectious source was identified through clinical evaluation or routine inflammatory markers. A comprehensive infectious workup was not performed because of the low pretest probability.

### 2.3 Further Clinical History

During further history taking, the patient's mother reported that he had been on a strict carbohydrate‐restricted diet (estimated < 20–30 g/day) for the week in preparation for an upcoming football tournament. This pattern of intake was consistent with a ketogenic diet rather than a moderate low‐carbohydrate restriction. The mother reported no prior symptoms of diabetes mellitus. However, she has a personal history of Type 1 diabetes mellitus (as noted above). First‐degree relatives of individuals with Type 1 diabetes have a significantly increased lifetime risk of developing the disease. No history of use of SGLT‐2 inhibitors, alcohol consumption, or other known precipitants of DKA was found.

### 2.4 Diagnosis

At the time of formal biochemical assessment, the patient had a blood glucose level of 198 mg/dL, high‐anion‐gap metabolic acidosis (pH 7.01, bicarbonate 14 mEq/L, anion gap 19 mEq/L), and 3+ urine ketones, fulfilling the biochemical criteria for euDKA. Although his blood glucose had transiently increased to 212 mg/dL during the preceding clinical deterioration, it had decreased to 198 mg/dL when the diagnostic evaluation was completed. These clinical features were consistent with previously undiagnosed diabetes mellitus and significant impairment of endogenous insulin secretion. Since autoantibody testing was not performed due to unavailability at our institution, this precluded confirmation of a specific type of diabetes. A low C‐peptide, acute presentation with ketosis, and a history of maternal Type 1 diabetes raised suspicion for severe insulin deficiency and possible Type 1 diabetes. However, without pancreatic autoantibody testing, a specific diabetes subtype could not be confirmed. Other phenotypes, including ketosis‐prone diabetes and antibody‐negative Type 1 diabetes, could not be excluded. The patient was admitted to the intensive care unit (ICU) for continued treatment.

### 2.5 Management

A modified DKA protocol was instituted in the ICU to account for the patient's euglycemic presentation. The main objectives were to suppress ketogenesis, correct the metabolic acidosis, and avoid hypoglycemia, as his blood glucose level was not significantly raised. Normal saline (0.9% NaCl) for intravenous (IV) fluid resuscitation was started within the first 2 h. Since his blood glucose was 198 mg/dL, he was also treated with 5% dextrose‐containing intravenous (IV) fluids to keep it within the target range of 150–200 mg/dL. An insulin infusion was initiated at 0.05 units/kg/h, a reduced rate selected because of the patient's near‐normal blood glucose and the high risk of hypoglycemia during continued insulin‐mediated suppression of ketogenesis. Dextrose‐containing fluids were administered concurrently to permit ongoing insulin therapy without inducing hypoglycemia [3, 6]. Potassium chloride (20 mEq/L) was added to the maintenance fluids with serial monitoring of serum potassium because insulin therapy can promote intracellular potassium shifts. An antiemetic was given to control vomiting. Frequent monitoring of blood glucose, serum electrolytes, ABG, and urine ketones was performed. Serum potassium remained within normal limits throughout, with no need for KCl dose adjustment. Based on these results, insulin infusion was continued, and IV fluids were titrated throughout the ICU stay to maintain blood glucose within the 150–200 mg/dL range. At the end of 28 h, the patient had an improved level of consciousness, an ABG pH of 7.29, and could tolerate oral fluids. Intravenous fluids were gradually tapered. At 36 h, the pH had normalized to 7.39, the anion gap had closed to 12 mEq/L, and bicarbonate had improved to 19 mEq/L. The patient's total ICU stay was approximately 36 h. The insulin infusion was successfully changed to subcutaneous insulin, and the patient was weaned from the ICU. The blood glucose was 212 mg/dL during the initial deterioration and subsequently decreased to 198 mg/dL at the time of formal biochemical evaluation and diagnosis (Table 2, Figure 1).

**TABLE 2:** Clinical timeline, laboratory trends, and treatment response.

Timepoint	pH	Bicarbonate (mEq/L)	Anion gap (mEq/L)	Urine ketones	Blood glucose (mg/dL)	Insulin infusion	Potassium (mEq/L)
0 h (ED arrival)	—	—	—	Not tested	164	None	—
~2 h (deterioration)	—	—	—	Positive (3+)	212	None	—
~2–4 h (euDKA diagnosis)	7.01	14	19	Positive (3+)	198	Started, 0.05 U/kg/h	3.9
~12 h	7.15	16	16	Positive (2+)	187	Continued, 0.05 U/kg/h	3.8
~20 h	7.22	17	14	Positive (1+)	179	Continued, 0.05 U/kg/h	4.0
~28 h	7.29	18	13	Trace	161	Continued, 0.05 U/kg/h	4.1
~36 h	7.39	19	12 (normal)	Negative	153	Switched to subcutaneous	4.2

**FIGURE 1:**
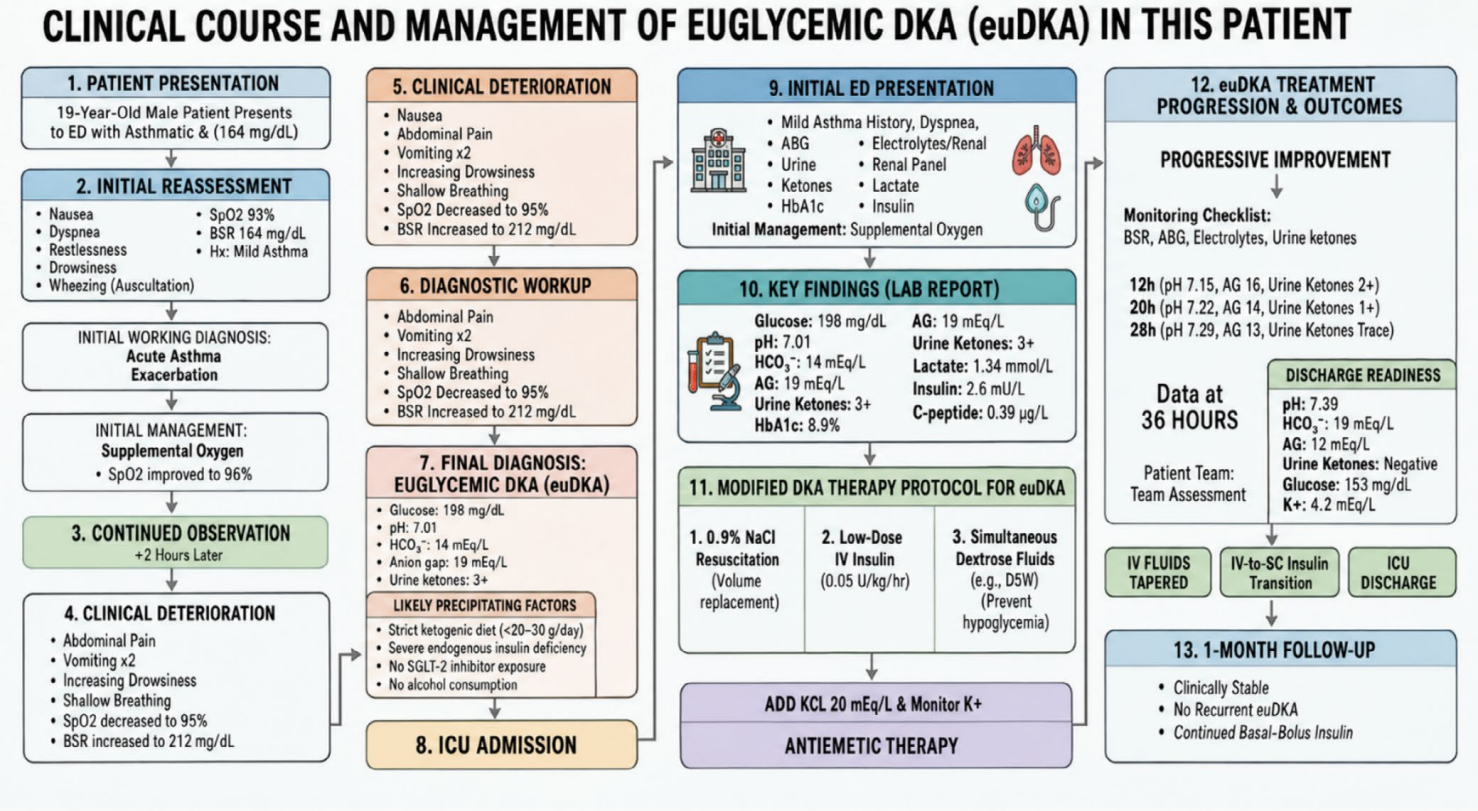
Clinical timeline and management flowchart for euglycemic diabetic ketoacidosis (euDKA). The figure was generated using Google Gemini (V.3.1 pro).

## 3 Results

At the 1‐month outpatient follow‐up, the patient remained clinically stable without recurrence of ketoacidosis. He continued basal–bolus insulin therapy with good adherence. However, diabetes classification by autoantibodies had not been done at the time of this report, and longer‐term follow‐up beyond 1 month was unavailable, so the short duration of follow‐up makes it difficult to assess long‐term glycemic control, trends in insulin use, or definitive diabetes classification.

## 4 Discussion

What makes our case significant is twofold: firstly, the presentation was initially mistaken for an acute asthma attack, and secondly, the precipitating factor was a carbohydrate‐restricted diet initiated by a young athlete who had completely undiagnosed diabetes. The mechanism underlying this patient's euDKA can be understood by considering the combination of a low endogenous insulin state and carbohydrate restriction. In a normal situation, insulin would inhibit lipolysis and hepatic ketogenesis, while glucagon would stimulate fatty acid oxidation and ketone body formation. When insulin is lacking, unchecked lipolysis increases the delivery of free fatty acids to the liver, where they are converted to acetoacetate and β‐hydroxybutyrate. At the same time, a low‐carbohydrate diet reduces the amount of glucose available to the body, and hepatic glycogen stores become depleted, further shifting energy metabolism toward fatty acid oxidation and ketogenesis. In people who have relatively normal pancreatic β‐cell function, this adaptive response is well controlled. Insulin deficiency was the central driver of this presentation: in our patient, insulin and C‐peptide levels were markedly low, implying marked insulin deficiency and resulting in hepatic ketone production exceeding peripheral utilization. This led to profound ketoacidosis despite a blood glucose level below the hyperglycemic threshold traditionally associated with DKA, resulting in a biochemical picture consistent with euDKA [3, 5, 7, 8]. The fasting C‐peptide level of 0.39 ng/mL (reference range 0.81–3.85 ng/mL) supported markedly reduced endogenous insulin secretion, consistent with significant insulin deficiency [8, 9].

Other metabolic and systemic causes of high‐anion‐gap metabolic acidosis were considered. Normal lactate and preserved renal function made lactic acidosis and uremia less likely, respectively, while no infectious source or supportive inflammatory findings were identified. Toxic ingestion was considered clinically unlikely, although salicylate and alcohol levels were not measured. Starvation ketosis was also considered given the strict carbohydrate restriction; however, the severity of acidosis, marked ketonuria, elevated HbA1c, and low C‐peptide favored DKA over uncomplicated nutritional ketosis.

The clinical presentation of shortness of breath, nausea, drowsiness, and wheeze in a patient with a known, spirometry‐confirmed history of asthma was initially very suggestive but also very confusing; however, wheezing on this presentation was assessed only by auscultation by the treating clinician and was not reconfirmed by spirometry or peak flow at the time of this acute episode. Respiratory compensation for metabolic acidosis in DKA classically manifests as deep, labored (Kussmaul) respirations, which can be mistaken for an asthma exacerbation in the appropriate clinical setting [2, 8], especially in patients with a preexisting asthma diagnosis. In this patient, however, his breathing was reported as shallow rather than deep and labored, an atypical pattern that likely contributed to the diagnostic delay by not prompting early suspicion of a metabolic rather than respiratory cause. The blood glucose was 164 mg/dL at presentation and 198 mg/dL at the time of clinical diagnosis, both below the 200 mg/dL threshold used to define euDKA in the 2024 consensus report. The transient improvement in oxygen saturation after supplemental oxygen reinforced the initial working diagnosis of asthma. This case highlights the importance of diagnostic anchoring, whereby a patient's overall condition may not improve or may worsen unexpectedly, so that the first diagnosis should be openly rethought.

The patient had been on a strict, ketogenic‐range carbohydrate restriction (estimated < 20–30 g/day) for 1 week before admission, hoping this would help his athletic performance for a football tournament. This is markedly stricter than the more general definition of a carbohydrate‐restricted eating pattern, commonly described as fewer than 130 g of carbohydrate per day or less than 26% of total daily energy intake, highlighting the severity of restriction in this case [7, 10]. In a severely insulin‐deficient individual, such a level of carbohydrate restriction can substantially reduce hepatic glycogen availability within a short period, promoting lipolysis and ketogenesis, which can rapidly become destructive in the absence of insulin control. In individuals with intact insulin secretion, physiological ketogenesis during carbohydrate restriction is generally regulated and does not typically progress to ketoacidosis. The risk is substantially greater in patients with severe insulin deficiency. These regulatory mechanisms may be insufficient in patients with severe insulin deficiency, including those with Type 1 or ketosis‐prone diabetes [5, 7]. Most reported cases of ketogenic diet‐induced euDKA have been in patients taking a ketogenic diet combined with an SGLT2 inhibitor [6], but Helvaci et al. [7] recently reported a case of euDKA resulting from a ketogenic diet alone in a patient with previously undiagnosed autoimmune diabetes, which bears striking similarities to our case in terms of the precipitant of the diet, beta‐cell dysfunction, and the atypical presentation in a young adult who had not previously been diagnosed with diabetes. Both our patient and the patient reported by Helvaci et al. [7] developed euDKA after carbohydrate restriction in the setting of previously unrecognized or untreated diabetes and severe endogenous insulin deficiency, without exposure to SGLT2 inhibitors. An additional diagnostic difficulty was that our case initially presented as an acute respiratory illness, just like the patient. Clinicians need to be aware that the increasing popularity of ketogenic and very low‐carbohydrate diets among adolescents and young adults, driven partly by social media trends and perceived athletic performance benefits, may pose a specific and under‐recognized metabolic risk in patients with undiagnosed or inadequately treated diabetes, even without other precipitants such as SGLT2 inhibitors.

The HbA1c of 8.9% indicated previously unrecognized chronic hyperglycemia over the preceding approximately 2–3 months before this admission, although the patient had never been diagnosed with diabetes. The carbohydrate‐restricted diet may have limited postprandial glucose excursions and osmotic diuresis, potentially contributing to the absence of classic hyperglycemic symptoms. The low C‐peptide level, acute presentation with ketosis, and maternal history of type 1 diabetes raised suspicion for autoimmune (type 1) diabetes, as seen in a similar case reported by Helvaci et al. [7]; however, autoantibody testing (e.g., GAD65, IA‐2, ZnT8, ICA) was not performed in our patient, and definitive classification could not be established, with ketosis‐prone diabetes and antibody‐negative Type 1 diabetes remaining possible differential classifications [11]. In the Helvaci et al. [7] case, islet cell antibody positivity (69.37 U/mL; reference < 28 U/mL) supported autoimmune diabetes in a clinically and biochemically similar presentation. The maternal history of Type 1 diabetes was clinically relevant, as first‐degree relatives of individuals with Type 1 diabetes have a substantially increased risk of developing the disease [12, 13].

Any young patient with a high‐anion‐gap metabolic acidosis (anion gap 19 mEq/L; normal < 12 mEq/L) should be suspected of DKA, even if the blood glucose is not elevated. The most important investigations were arterial blood gas (pH 7.01, HCO_3_ 14 mEq/L), positive urine ketones, and HbA1c. A normal lactate level made significant lactic acidosis unlikely, while normal renal function argued against uremia as a major contributor to the anion gap. It is worth noting that the urine dipstick used for the standard urinalysis detects only acetoacetate, not β‐hydroxybutyrate, which accounts for about 75%–85% of circulating ketone bodies in DKA. Where available, serum β‐hydroxybutyrate (BHB) (normal < 0.6 mmol/L; almost always > 3 mmol/L in DKA) is the preferred test; urine dipstick alone tends to underestimate ketosis [14]. Recent international consensus recommendations emphasize that DKA may occur in the absence of marked hyperglycemia and specifically recognize euDKA as an important diagnostic entity [3].

In euDKA, dextrose‐containing fluids may need to be administered concurrently with insulin to permit continued suppression of ketogenesis while avoiding hypoglycemia. Because the blood glucose was already low, dextrose‐containing fluids were initiated concurrently with insulin to permit continued insulin administration while avoiding hypoglycemia, consistent with current consensus recommendations [3]. Potassium supplementation was continued with serial monitoring, and serum potassium remained within normal limits throughout the ICU stay. It appears that our patient fully recovered within 36 h, which may be attributed to the short period during which he restricted carbohydrates prior to presentation.

## 5 Conclusion

euDKA is a diagnostic challenge and depends on a high index of clinical suspicion, especially for young patients without a known history of diabetes mellitus. Normal or near‐normal blood glucose does not exclude DKA. This case illustrates how a very low‐carbohydrate diet can precipitate profound ketoacidosis, resulting in life‐threatening euDKA in an individual with previously undiagnosed diabetes with severe endogenous insulin deficiency, and can similarly manifest itself as acute asthma. If, after the first treatment, a patient is not improving as expected, the physician's differential diagnosis should be expanded. If a patient's metabolic state deteriorates, he or she becomes less alert or has unusual breathing patterns, an early arterial blood gas or urinalysis for ketones should be performed, even if glucose is close to the normal range. While this single case cannot establish generalizable treatment protocols, it reinforces the importance of considering DKA in atypical respiratory presentations and using concurrent dextrose‐containing fluids when necessary to permit continued insulin therapy while minimizing hypoglycemia in euDKA.

## 6 Limitations

Several limitations in this case should be recognized. We did not have a facility to measure serum ketone (beta‐hydroxybutyrate) levels, and we were only able to diagnose ketosis by urine ketone testing, which is less precise. Salicylate and blood alcohol were not measured, but toxic ingestion was not suspected based on clinical history. During this admission, blood tests for autoimmune antibodies were not performed to establish the type of diabetes. Finally, further evaluation for diabetes classification, including pancreatic autoantibody testing, was planned but was not available at our institution. As a single case report, our findings and management approach may not be generalizable to all patients with euDKA, and larger case series or studies are needed to confirm these observations. These limitations should be considered when interpreting the diagnosis, diabetes classification, and treatment course described in this single case.

## Author Contributions

**Faizan E Mustafa:** conceptualization, methodology, investigation, data curation, formal analysis, resources, writing – original draft, writing – review and editing, visualization, project administration. **Sarim Hassan Shahab:** writing – original draft, writing – review and editing. **Basit Ali:** writing – review and editing, writing – original draft. **Aleena Majeed:** writing – original draft, writing – review and editing. **Fazal Habib:** writing – review and editing. **Muhammad Sajjad:** writing – review and editing.

## Funding

The authors have nothing to report.

## Ethics Statement

The authors have nothing to report.

## Consent

Written informed consent was obtained from the patient for publication of this case report and accompanying images.

## Conflicts of Interest

The authors declare no conflicts of interest.

## Data Availability

Data sharing is not applicable to this article as no datasets were generated or analyzed during this study.
